# Transcription factor-based gene therapy to treat glioblastoma through direct neuronal conversion

**DOI:** 10.20892/j.issn.2095-3941.2020.0499

**Published:** 2021-08-15

**Authors:** Xin Wang, Zifei Pei, Aasma Hossain, Yuting Bai, Gong Chen

**Affiliations:** 1 Department of Biology, Huck Institutes of Life Sciences, Pennsylvania State University, University Park, PA 16802, USA; 2 GHM Institute of CNS Regeneration, Jinan University, Guangzhou 510632, China

**Keywords:** Glioblastoma, neuronal conversion, transcription factors, NeuroD1, neurogenin-2, Ascl1

## Abstract

**Objective:**

Glioblastoma (GBM) is the most prevalent and aggressive adult primary cancer in the central nervous system. Therapeutic approaches for GBM treatment are under intense investigation, including the use of emerging immunotherapies. Here, we propose an alternative approach to treat GBM through reprogramming proliferative GBM cells into non-proliferative neurons.

**Methods:**

Retroviruses were used to target highly proliferative human GBM cells through overexpression of neural transcription factors. Immunostaining, electrophysiological recording, and bulk RNA-seq were performed to investigate the mechanisms underlying the neuronal conversion of human GBM cells. An *in vivo* intracranial xenograft mouse model was used to examine the neuronal conversion of human GBM cells.

**Results:**

We report efficient neuronal conversion from human GBM cells by overexpressing single neural transcription factor Neurogenic differentiation 1 (NeuroD1), Neurogenin-2 (Neurog2), or Achaete-scute homolog 1 (Ascl1). Subtype characterization showed that the majority of Neurog2- and NeuroD1-converted neurons were glutamatergic, while Ascl1 favored GABAergic neuron generation. The GBM cell-converted neurons not only showed pan-neuronal markers but also exhibited neuron-specific electrophysiological activities. Transcriptome analyses revealed that neuronal genes were activated in glioma cells after overexpression of neural transcription factors, and different signaling pathways were activated by different neural transcription factors. Importantly, the neuronal conversion of GBM cells was accompanied by significant inhibition of GBM cell proliferation in both *in vitro* and *in vivo* models.

**Conclusions:**

These results suggest that GBM cells can be reprogrammed into different subtypes of neurons, leading to a potential alternative approach to treat brain tumors using *in vivo* cell conversion technology.

## Introduction

Glioblastoma (GBM) is a type of tumor that arises from unchecked proliferation of glial cells in the brain and spinal cord^1^. It is the most invasive primary malignant cancer in adults. In 2019, an estimated 23,820 new cases of GBM and 17,760 deaths were reported in the United States^2^. Therapeutic approaches for glioblastoma treatment are largely hampered by its active proliferation, highly invasive nature, and heterogeneity^3,4^. Current therapeutic approaches, including immunotherapy using CART or PD-1/PD-L1^5–7^, try to kill or remove glioma cells entirely from the body, but this is very difficult to achieve. Therefore, it is urgent to identify alternative therapeutic approaches for GBM.

Our laboratory, together with other laboratories, pioneered an *in vivo* cell conversion approach to directly convert brain internal glial cells into neurons through ectopic expression of neural transcription factors^8–12^. Because glioma cells originate from proliferative glial cells, we hypothesized that it might be possible to convert glioma cells into neurons. Indeed, several studies have reported some preliminary success in this conversion^13–18^, but the underlying mechanisms remain largely unknown.

In the present study, we characterized the cell conversion capabilities of 3 different neural transcription factors, including Neurog2, NeuroD1, and Ascl1. We showed that each individual factor efficiently converted GBM cells into neuron-like cells. The converted cells not only displayed pan-neuronal markers, but also fired action potentials, a typical electrophysiological property of neurons. Transcriptome analyses further confirmed the upregulation of neuronal genes by neural transcription factors after their overexpression in GBM cells. Moreover, neuronal conversion of GBM cells effectively arrested cell proliferation both *in vitro* and *in vivo*. These studies suggest that transcription factor-based gene therapy may be a potential alternative approach to treat GBM.

## Materials and methods

### Cell culture

Human GBM cell lines were purchased from Sigma-Aldrich (St. Louis, MO, USA) (U251) or ATCC (Manassas, VA, USA) (U118). U251 cells were cultured in GBM culture medium, which included MEM (Gibco, Gaithersburg, MD, USA), 0.2% penicillin/streptomycin (Gibco), 10% fetal bovine serum (FBS; Gibco), 1 mM sodium pyruvate (Gibco), 1% non-essential amino acids (Gibco), and 1× GlutMAX (Gibco). U118 cells were cultured in medium containing DMEM (Gibco), 10% FBS, and 1% penicillin/streptomycin.

Human astrocytes were purchased from ScienCell (San Diego, CA, USA). Human astrocytes were cultured in human astrocyte medium, which included DMEM/F12 (Gibco), 10% FBS, 3.5 mM glucose (Sigma-Aldrich), and 0.2% penicillin/streptomycin, supplemented with B27 (Gibco), N2 (Gibco), 10 ng/mL fibroblast growth factor 2 (Invitrogen, Carlsbad, CA, USA), and 10 ng/mL epidermal growth factor (Invitrogen).

For subcultures, the cells were trypsinized using 0.25% trypsin (Gibco) or TrypLE Select (Invitrogen), centrifuged for 5 min at 800 rpm, resuspended, and plated in corresponding culture medium with a split ratio of approximately 1:4. The cells were maintained at 37 °C in humidified air with 5% CO_2_.

### Reprogramming human GBM cells into neurons

U251 cells were seeded in poly-D-lysine-coated coverslips in 24-well plates at least 12 h before the virus infection at a density of 10,000 cells per coverslip. GFP, Neurog2, NeuroD1, or Ascl1 retrovirus was added to GBM cells together with 8 µg/mL Polybrene (Santa Cruz Biotechnology, Santa Cruz, CA, USA). The culture medium was completely replaced by neuronal differentiation medium (NDM) the next day to aid in neuronal differentiation and maturation. NDM included DMEM/F12 (Gibco), 0.4% B27 supplement (Gibco), 0.8% N2 supplement (Gibco), 0.2% penicillin/streptomycin, 0.5% FBS, vitamin C (5 µg/mL, Selleck Chemicals, Houston, TX, USA), Y27632 (1 µM; Tocris Bioscience, Bristol, UK), glial cell-derived neurotrophic factor (GDNF; 10 ng/mL; Invitrogen), brain-derived neurotrophic factor (BDNF; 10 ng/mL; Invitrogen), and neurotrophin 3 (NT3; 10 ng/mL; Invitrogen). The cells were maintained at 37 °C in humidified air with 5% CO_2_.

### *In vivo* neuronal conversion of human GBM cells

*In vivo* neuronal conversion of human GBM cells was conducted using Rag1 knockout (KO) immunodeficient mice (B6.129S7-Rag1^tm1Mom^/J; #002216; The Jackson Laboratory, Bar Harbor, ME, USA). Half a million (5 × 10^5^) U251 human GBM cells were transplanted into the striatum of Rag1 KO mouse brains using a stereotaxic device (Hamilton, Las Vegas, NV, USA). Retroviruses expressing Neurog2-GFP or green fluorescent protein (GFP) alone with similar titers were injected intracranially at the same time and location. Mouse brains were harvested and sliced at 1, 2, 4, and 8 weeks after injection. Immunostaining for brain slice sections was the same as cultured cells. The experimental protocols were approved by The Pennsylvania State University IACUC (IACUC # 47890).

### Next-generation sequencing and data analysis

RNA was extracted using the NucleoSpin® RNA kit (Macherey-Nagel, Duren, Germany) following the manufacturer’s protocols. RNA samples came from 3 batches of U251 GBM cells overexpressing GFP alone or with Neurog2-GFP, or Ascl1- GFP, for a total of 9 samples. Quality checks of RNA samples, mRNA enrichment, library construction, and next-generation sequencing (single-end, 50 bp; Hiseq 3000 platform; Illumina, San Diego, CA, USA) were performed at the UCLA Technology Center for Genomics and Bioinformatics (Los Angeles, CA, USA). The raw data (fastq files) were checked using FastQC (v. 0.11.3) with default settings^19^. The read alignment (against the hg38 human reference genome) was performed using BWA-MEM (v. 0.7.17-r1188) and summarized using featureCounts (v. 1.5.0)^20,21^. Differential expression analysis was processed using DESeq2 (v. 1.16.1)^22^. Differentially-expressed genes (DEGs) in the Neurog2 or Ascl1 overexpression group were defined with adjusted values of *P* < 0.05 and fold change > 2, when compared with the GFP group using DESeq2. Heat maps were created using R console as previously described^23^. Gene ontology was analyzed using the Gene Ontology Consortium (http://geneontology.org/). Gene enrichment analysis was conducted using gene set enrichment analysis (GSEA) software^24^. The raw fastq files and the normalized read count file were uploaded in Gene Expression Omnibus (GEO, GSE161534). Other methods are described in the Supplementary Materials.

## Results

### Efficient neuronal conversion of human GBM cells

**Figure 1:**
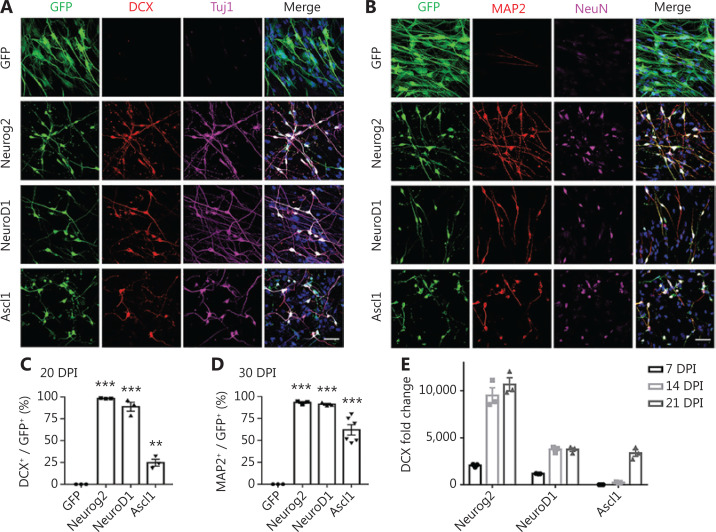
Neural transcription factor Neurog2, NeuroD1, or Ascl1 converts human glioblastoma cells into neurons. (A, B) Retroviral expression of Neurog2, NeuroD1, or Ascl1 in U251 human glioblastoma cells led to conversion of a large number of neuronal cells compared to the green fluorescent protein (GFP)-treated control group (top row). Neurog2-, NeuroD1-, or Ascl1-converted cells were immunopositive for immature neuronal markers (A; DCX, red; Tuj1, magenta) at 20 days post-infection (dpi), and mature neuronal markers (B; MAP2, red; NeuN, magenta) at 30 dpi. Scale bars, 50 μm. (C, D) Quantitative analyses of the conversion efficiency at 20 dpi (C) and 30 dpi (D). ^**^P < 0.01; ^***^P < 0.001; one-way analysis of variance followed by Dunnett’s test; *N* ≥ 200 cells from triplicate or more cultures. (E) Transcriptional activation of DCX during conversion revealed by real-time qPCR. Data were normalized to control GFP samples and represented as the mean ± SEM. *N* = 3 batches.

We recently reported that ectopic expression of a single neural transcription factor, NeuroD1, efficiently converted astrocytes into neurons^9,25–31^. NeuroD1, together with Neurog2 and Ascl1, belongs to the basic helix-loop-helix (bHLH) family of neural transcription factors, and plays critical roles in the induction of neural differentiation during early brain development^11,32–36^. Following our successful neuronal conversion of glial cells, we further tested the possibility of converting glioma cells into neurons using neural transcription factors. We first characterized the GBM cell lines used in this study, which exhibited typical astroglial signatures with rare contamination of progenitors or stem cells (**Supplementary Figure S1**). They also showed a high proliferation rate (Ki67, **Supplementary Figure S1**). To target the highly proliferative GBM cells, we constructed retroviral vectors that efficiently targeted dividing cells to overexpress Neurog2 (CAG::Neurog2-IRES-eGFP), NeuroD1 (CAG::NeuroD1-IRES-eGFP), or Ascl1 (CAG::Ascl1-IRES-eGFP) in GBM cells. Retrovirus expressing GFP alone was used as a control (**Figure 1A**). After overexpressing Neurog2 or NeuroD1 in GBM cells (U251; Sigma-Aldrich), we found that the majority of virally-infected GBM cells had a neuronal morphology, and showed immature neuronal markers such as doublecortin (DCX) and β3-tubulin (Tuj1) at 20 days post-infection (dpi), but only a small proportion of Ascl1-infected GBM cells were converted into neuron-like cells (**Figure 1A**). At 30 dpi, the mature neuronal markers, MAP2 and NeuN, were both detected among Neurog2-, NeuroD1-, or Ascl1-infected cells (**Figure 1B**). Quantitative analyses showed that among the 3 bHLH factors, the conversion efficiency was highest for Neurog2 (98.2% ± 0.3%), followed by NeuroD1 (88.7% ± 5.2%), and lowest for Ascl1 (24.6% ± 4.0%) at 20 dpi (**Figure 1C**, *N* = 3 repeats). At 30 dpi, the conversion efficiency was 93.2% ± 1.2% for Neruog2, 91.2% ± 1.1% for NeuroD1, and 62.1% ± 5.9% for Ascl1 (**Figure 1D**, *N* > 3 repeats). Besides the immunostaining approach, neuronal conversion of GBM cells was further investigated using real-time quantitative PCR (RT-qPCR) analysis to determine the time course of transcriptional changes induced by neural transcription factors. Among Neurog2-infected GBM cells, we detected a significant increase in the transcriptional activation of DCX (> 2,000-fold increase) at 7 dpi, which further surged to a 10,000-fold change at 14–21 dpi (**Figure 1E**; Neurog2). NeuroD1-infected GBM cells also showed a significant increase of DCX (> 1,000-fold increase) at 7 dpi and over 3,000-fold increase by 14–21 dpi (**Figure 1E**; NeuroD1). Notably, Ascl1-infected GBM cells did not show significant activation of DCX until 21 dpi (**Figure 1E**, Ascl1), consistent with the low conversion efficiency of Ascl1 when assessed with DCX immunostaining. The low conversion efficiency by Ascl1 was not due to low expression of Ascl1 in GBM cells, because we confirmed the overexpression of Neurog2, NeuroD1, or Ascl1 using immunohistochemistry (**Supplementary Figure S2A**), as well as by using RT-qPCR (**Supplementary Figure S2B**), which actually found an increase of Ascl1 mRNA level among Ascl1-infected GBM cells (20 dpi). We further performed immunostaining of the immature neuronal markers, DCX and Tuj1, at 6 dpi (**Supplementary Figure S3A–S3C**) and found that consistent with our RT-qPCR results, Neurog2 showed the highest conversion efficiency, while Ascl1 showed the lowest conversion efficiency. Therefore, the 3 different bHLH family neural transcription factors had different potencies in converting glioma cells into neurons. It is worth noting that despite a mild initial apoptosis caused by retroviral infection, there was no significant cell apoptosis observed during the conversion process (**Supplementary Figure S4**).

### Characterization of the converted neurons from human GBM cells

**Figure 2:**
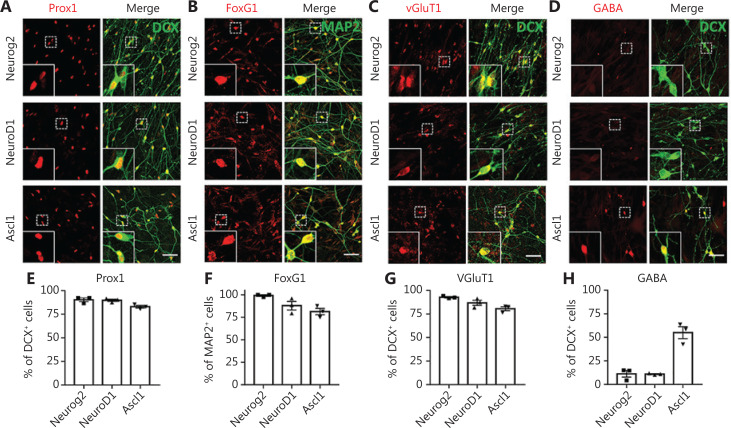
Characterization of the converted neurons from human glioblastoma (GBM) cells. (A–D) Representative images showing the immunostaining of neuronal subtype markers in the converted neurons from U251 human GBM cells. Most of the Neurog2-, NeuroD1-, and Ascl1-converted neurons (DCX or MAP2, green) were immunopositive for hippocampal neuron marker Prox1 (red in A), forebrain neuron marker FoxG1 (red in B), and glutamatergic neuron marker VGluT1 (red in C). Note that there were many GABA^+^ neurons (red in D) converted by Ascl1 instead of Neurog2 or NeuroD1. (E–H) Quantitative analyses of the converted neuron subtypes. Samples were at 20 dpi. Scale bars, 50 μm. Data are represented as the mean ± SEM. *N* ≥ 200 cells from triplicate cultures.

We next characterized the converted neurons from GBM cells using neuronal markers expressed in different brain regions. We found that the majority of converted cells were immunopositive for hippocampal granule neuron marker Prox1 (**Figure 2A**; quantified in **Figure 2E**: Neurog2, 90.4% ± 1.9%; NeuroD1, 89.9% ± 1.2%; Ascl1, 83.0% ± 1.4%; Prox1^+^/DCX^+^ cells), and forebrain marker FoxG1 (**Figure 2B**; quantified in **Figure 2F**: Neurog2, 99.2% ± 0.8%; NeuroD1, 87.9% ± 4.8%; Ascl1, 81.3% ± 3.6%; FoxG1^+^/MAP2^+^ cells). However, in contrast to the astrocyte-converted neurons in our previous studies^8,9,37,38^, few neurons converted from GBM cells expressed cortical neuron marker Ctip2 or Tbr1 (**Supplementary Figure S5A–S5B**). These results suggested that intrinsic imprinting of human glioblastoma cells may be different from astroglial cells, and may influence the cell fate after conversion. To directly test this hypothesis, we performed side-by-side comparisons with neurons converted from human astrocytes (HA1800; ScienCell). The majority of the Neurog2-, NeuroD1-, or Ascl1-converted neurons from human astrocytes were positive for FoxG1 and Prox1, with a significant proportion immunopositive for Ctip2 (**Supplementary Figure S6A–S6B**). Neurons converted from GBM cells therefore shared some common properties with the neurons converted from astrocytes, but differed in the specific neuronal subtypes.

Next, we characterized the converted neuronal subtypes according to the neurotransmitters released, in particular glutamatergic and GABAergic neurons, which are the principal excitatory and inhibitory neurons in the brain, respectively. Most Neurog2-, NeuroD1-, and Ascl1-converted cells were immunopositive for the glutamatergic neuron marker, VGluT1 (**Figure 2C**; quantified in **Figure 2G**: Neurog2, 92.8% ± 0.7%; NeurD1, 86.9% ± 2.7%; Ascl1, 80.6% ± 2.1%; VGluT1^+^/DCX^+^ cells). The majority of Neurog2- and NeuroD1-converted cells were immunonegative for GABA (**Figure 2D**; quantified in **Figure 2H**: Neurog2, 11.1% ± 3.8%; NeuroD1, 8.6% ± 2.5%; GABA^+^/DCX^+^ cells). In contrast, roughly half of the Ascl1-converted cells were GABA-positive neurons (**Figure 2D**; quantified in **Figure 2H**: Ascl1, 55.0% ± 6.4%, GABA^+^/DCX^+^ cells), reflecting the differences among different neuronal conversion factors. We next tested several combinations of transcription factors including NeuroD1 and Ascl1, but did not notice any further improvements regarding the conversion efficiency or neuronal subtypes (**Supplementary Figure S7**).

In summary, the majority of the Neurog2- or NeuroD1-converted neurons were forebrain glutamatergic neurons, while Ascl1 showed a trend for GABAergic neuron generation. Therefore, expression of different transcription factors will have significant influence on the converted neuronal, subtypes.

### Fate changes of GBM cells to neurons induced by Neurog2 overexpression

**Figure 3:**
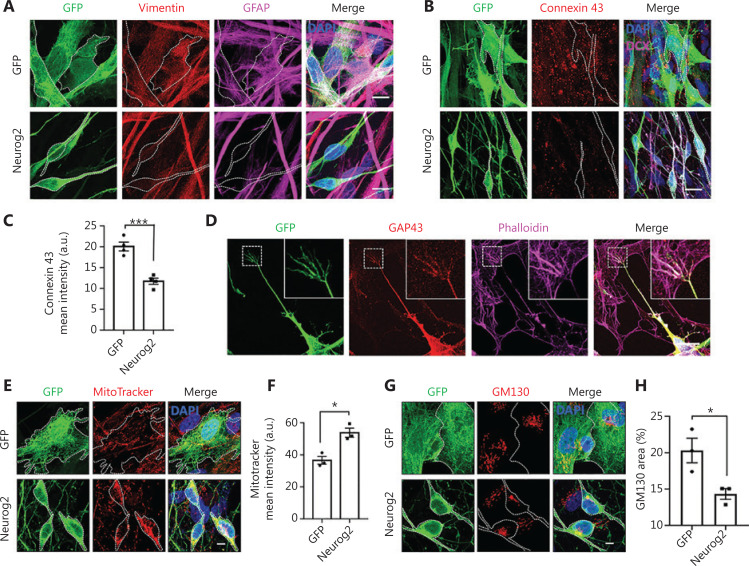
Change from glioblastoma (GBM) cells to neurons induced by Neurog2 overexpression. (A) Downregulation of astrocyte markers vimentin (red) and GFAP (magenta) in Neurog2-converted neurons (bottom row) compared with the control green fluorescent protein (GFP)-expressing U251 cells (top row). Samples were at 20 dpi. (B) Representative images showing the gap junctions (Connexin 43, red) in U251 GBM cells expressing GFP alone (top row) or Neurog2 (bottom row) at 20 dpi. (C) Quantitative data showing a significant reduction of the Connexin 43 mean intensity in Neurog2-converted neurons compared with control cells. Samples were at 20 dpi. *N* ≥ 60 from triplicate cultures. (D) Representative images illustrating the fingerlike filopodia of a growth cone depicted by growth cone marker Growth Associated Protein 43, red) and filamentous actin probe Phalloidin (magenta) in Neurog2-converted neurons. Samples were at 6 dpi. (E–H) Distribution and morphological changes of mitochondria (MitoTracker, red in E) and the Golgi apparatus (GM130, red in G) during Neurog2-induced neuronal conversion in U251 cells. Quantitative data showing the MitoTracker mean intensity (F) and the normalized GM130 covered area (H). Samples were at 30 dpi. *N* ≥ 150 from triplicate cultures. Scale bars, 20 μm in (A), (B), and (D); 10 μm in (E) and (G). Data are represented as the mean ± SEM and analyzed by Student’s *t*-test. ^*^P < 0.05; ^***^P < 0.001.

Because Neurog2 yielded the fastest and most efficient neuronal conversion in GBM cells, we further investigated the Neurog2-induced conversion process in detail. Previous studies reported that the astrocyte marker GFAP and the epithelial-mesenchymal transition marker vimentin were both highly expressed in human U251 GBM cells^39,40^. After Neurog2 overexpression for 20 days, both GFAP and vimentin were downregulated in converted cells compared to the control (**Figure 3A**, **Supplementary Figure S8**). The gap junction marker, Connexin 43, was also downregulated after Neurog2 overexpression (**Figure 3B**; quantitation of the Connexin 43 intensity in **Figure 3C**: GFP control, 19.4 ± 0.7 a.u.; Neurog2, 11.6 ± 0.8 a.u.; at 20 days after infection), which was consistent with neurons having less gap junctions compared with glial cells^41^. Notably, we observed typical growth cone structures among the Neurog2-converted neurons (**Figure 3D**), which showed fingerlike filopodia labeled by filamentous actin (F-actin) when probed with Phalloidin and the growth cone marker, GAP43 (**Figure 3D**).

Neurons are highly polarized, which differ from GBM cells. We wondered what would happen to cellular organelles such as mitochondria and the Golgi apparatus during cell conversion, given their important roles in maintaining cellular functions and homeostasis. Mitochondria are known to locate in areas with high energy demands. In GBM cells, mitochondria were distributed in the cytoplasm without obvious polarization; whereas after Neurog2-induced conversion, mitochondria showed a significant change in the distribution pattern, with concentrated localization in the soma surrounding the nucleus (**Figure 3E**). Compared to the control at 30 dpi, the mean intensity of mitochondria significantly increased in the converted neurons (**Figure 3F**). Similarly, the distribution of the Golgi apparatus also showed a significant change between Neurog2-converted neurons and control GBM cells (**Figure 3G**). Compared to the control group, the normalized area of the Golgi apparatus was much smaller in Neurog2-converted neurons (**Figure 3H**). However, autophagy activity was found to be comparable between the Neurog2-converted and control cells (**Supplementary Figure S9A– S9C**). Together, the results showed that subcellular distribution patterns of cellular organelles underwent a significant change during the cell conversion process from GBM cells to neurons.

### Functional analyses of neurons converted from human GBM cells

**Figure 4:**
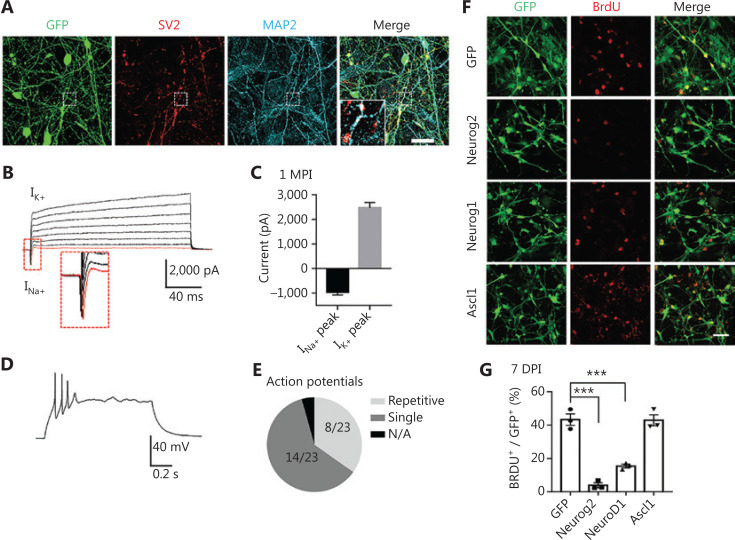
Functional analyses of human glioblastoma (GBM) cell-converted neurons and the cell proliferation test. (A) Robust synaptic puncta (SV2, red) were detected along the dendrites (MAP2, cyan) in Neurog2-converted neurons from U251 human GBM cells. Samples were at 30 dpi. Scale bars, 20 μm. (B, C) Representative traces (B) showing Na^+^ and K^+^ currents recorded from Neurog2-converted neurons, with quantitative analyses shown in (C). Samples were at 30 dpi. *N* ≥ 20 from triplicate cultures. (D, E) Whole-cell patch clamp recordings revealed action potentials firing from Neurog2-converted neurons (D), with a pie chart indicating the fraction of cells firing single (dark grey, E), repetitive (light grey, E), or no action potentials (black, E). Samples were at 30 dpi. *N* ≥ 20 from triplicate cultures. (F) Representative images examining cell proliferation using bromodeoxyuridine (BrdU) immunostaining (red) in U251 human glioblastoma cells expressing green fluorescent protein, Neurog2, NeuroD1, or Ascl1 (green). Cell cultures were incubated in 10 mM BrdU for 24 h before immunostaining at 7 dpi. Scale bars, 50 μm. (G) Quantitative analyses of the proliferative cells (BrdU^+^ cells/total GFP^+^ infected cells) at 7 dpi. Data were analyzed by one-way analysis of variance followed with Dunnett’s test. ^***^P < 0.001; *N* ≥ 200 cells from triplicate cultures. Data are represented as the mean ± SEM.

A critical factor for testing neuronal conversion is whether the GBM cell-converted cells form neuronal connections and exhibit functional properties. We investigated the capability of the Neurog2-converted cells to form synapses by performing immunostaining for the SV2 synaptic vesicle marker. We detected intensive synaptic puncta along MAP2-labeled dendrites in the Neurog2-converted neurons at 30 dpi (**Figure 4A**). Patch-clamp recordings showed significant sodium and potassium currents in the converted neurons (**Figure 4B–4C**, 30 dpi). The majority of Neurog2-converted neurons fired single action potentials (14 out of 23), while a subset of the converted neurons (8 out of 23) fired multiple action potentials (**Figure 4D–4E**). However, no spontaneous synaptic events were recorded in the Neurog2-converted neurons at 30 dpi, suggesting that the converted neurons may still be immature, or perhaps the surrounding glioma cells inhibited neuronal functions. Together, these results indicated that human GBM cells could be reprogrammed into neuron-like cells, but with partial neuronal functions when surrounded by glioma cells.

### Arrest of cell proliferation through cell conversion

Neurons are terminally differentiated non-proliferating cells. Neuronal reprogramming may therefore be a promising strategy to control cancer cell proliferation. To test this hypothesis, we examined cell proliferation at the early stage of conversion. GBM cells at 7 days after viral infection were incubated with 10 mM BrdU for 24 h to label the proliferating cells (**Figure 4F**). Quantification of the percentage of BrdU positive cells showed that compared with the GFP control, the proliferation of Neurog2- and NeuroD1-infected cells significantly decreased (**Figure 4G**; GFP, 64.8% ± 4.1%; Neurog2, 11.9% ± 2.9%; NeuroD1, 24.5% ± 2.4%; 7 dpi). However, the proliferation of Ascl1-converted cells remained active at 7 dpi (**Figure 4F**; quantitated in **Figure 4G**; Ascl1, 54.6% ± 1.2%), possibly due to a slow action of Ascl1 in GBM cells (**Figure 1A–1D**, **S2A– S2C**). Overall, the proliferation rate of GBM cells was significantly decreased after overexpression of Neurog2 or NeuroD1, consistent with the fast-converting speed of Neurog2 and NeuroD1 after infecting GBM cells. Together, these results suggest that in addition to neuronal conversion, ectopic expression of neuronal transcription factors may also be a promising approach to control GBM cell proliferation.

We also investigated biomarkers of glioblastoma with or without neuronal conversion. Notably, the EGFR and IL13Ra2 glioma markers were clearly detected after neuronal conversion at 20 days after Neurog2 infection (**Supplementary Figure S10A–S10D**)^42–44^, suggesting that the newly converted neurons may still have retained characteristics of GBM cells, at least for the early time period after conversion.

### Transcriptome analyses of human GBM cell conversion

To identify the underlying mechanism of the glioblastoma cell-to-neuron conversion process, we performed RNA-sequencing (RNA-seq) and transcriptome analyses of GBM cells after Neurog2 or Ascl1 overexpression, with GFP alone serving as the control group (3 replicates for each group). The RNA samples were prepared at 5 DPI to capture the early responses and potential direct targets of neural transcription factors in the early stages of conversion.

**Figure 5:**
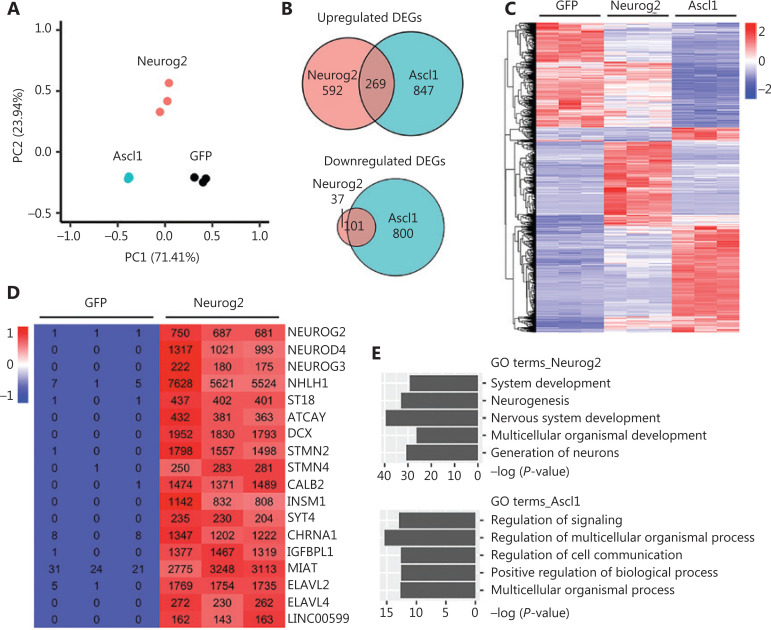
Transcriptome analyses of human glioblastoma (GBM) cells with Neurog2 or Ascl1 overexpression. (A) Principal component analysis comparing the global gene expression profiles of U251 human GBM cells infected by Neurog2, Ascl1, or control green fluorescent protein (GFP) retroviruses. Samples were collected at 5 DPI. *N* = 3 biological replicates for each group. Note that Ascl1 samples are very much clustered together. (B) Venn diagrams showing the number of differentially-expressed genes (DEGs) (adjusted *P* < 0.05, fold change > 2, compared with control GFP samples) in the Ascl1 or Neurog2 groups. (C) A heat map with hierarchical clustering showing the DEGs (adjusted *P* < 0.05, fold change > 2, total 2,612) in response to Ascl1 or Neurog2 overexpression in U251 cells. The color was scaled within each row. Normalized read count values are presented. (D) A heat map illustrating that the top upregulated DEGs in Neurog2 samples were closely related with neurogenesis. The color was scaled within each row. (E) Gene Ontology terms of the upregulated DEGs (adjusted *P* < 0.01, fold change > 3, compared with control GFP samples) in response to Neurog2 or Ascl1 overexpression.

Principal component analysis (PCA) showed a clear segregation of the global gene expression profiles of different groups (**Figure 5A**). Pair-wise differential expression analyses showed a total of 2,612 DEGs (fold change > 2; adjusted *P* < 0.05) identified in the Ascl1 (2,017 DEGs) or Neurog2 (999 DEGs) groups, when compared with control GFP samples (**Figure 5B–5C**). Notably, while both Ascl1 and Neurog2 belong to the bHLH family of neural transcription factors, only a small number of DEGs (14%; 370 out of 2,612 DEGs, **Figure 5B–5C**) were commonly regulated by both Ascl1 and Neurog2 among the infected GBM cells. We then identified the top upregulated DEGs as potential downstream targets of the transcription factors. Most of the top upregulated DEGs of Neurog2 were closely related with neurogenesis (**Figure 5D**). Some were well-known neural transcription factors including NEUROG3, NEUROD4, NHLH1, and ST18. Neuronal genes were also strongly upregulated by Neurog2 such as *DCX* and calbindin 2 (*CALB2*), consistent with our immunostaining results. We also identified several interesting molecular mediators associated with microRNA and RNA regulations, such as ELAV like RNA binding protein 2 (ELAVL2), ELAV like RNA binding protein 4 (ELAVL4), and long intergenic non-protein coding RNA 599 (LINC00599). In contrast, the top DEGs induced by Ascl1 were not specific to the nervous system, but involved developmental regulations, such as bone gamma-carboxyglutamate protein (BGLAP), calcium/calmodulin dependent protein kinase II beta (CAMK2B), and down syndrome cell adhesion molecule (DSCAM) (**Supplementary Figure S11**). Consistent with these findings, most of the Gene Ontology (GO) terms enriched in the Neurog2-upregulated DEGs (adjusted *P* < 0.01, fold change > 3, compared with GFP samples) involved neurogenesis and nervous system development (**Figure 5E**). In contrast, more general GO terms were found in Ascl1-upregulated DEGs (adjusted *P* < 0.01, fold change > 3, compared with GFP samples), such as the regulation of signaling and multicellular organismal processes (**Figure 5E**). Together, these results implied divergent transcriptome changes in response to Neurog2 or Ascl1 overexpression in human GBM cells.

**Figure 6:**
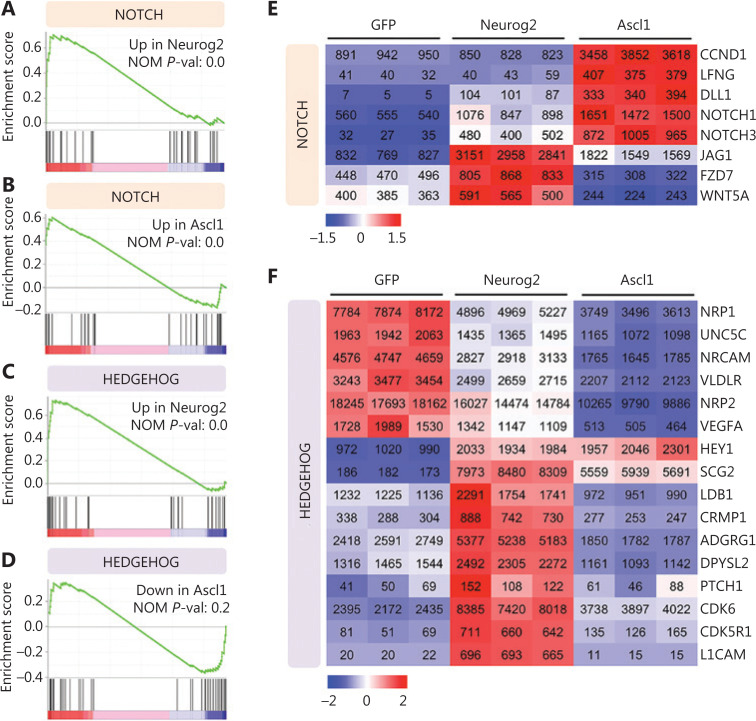
Signaling pathway changes in response to Neurog2 or Ascl1 overexpression in human glioblastoma (GBM) cells. (A–D) Gene set enrichment analysis of RNA-seq data showing that the Notch signaling pathway was activated in response to Neurog2 (A) or Ascl1 (B) overexpression in U251 human glioblastoma cells. In contrast, the Hedgehog signaling was significantly upregulated by Neurog2 (C) but not Ascl1 (D). (E, F) Heat maps showing the leading-edge subsets of genes (> 100 normalized read counts at least in 1 sample) corresponding to the Notch (E) or the Hedgehog (F) signaling pathway shown in (A–D). The color was scaled within each row. Normalized read count values are presented.

We then investigated the signaling pathways regulated by Neurog2 versus Ascl1. GSEA showed that Neurog2 and Ascl1 both activated the Notch signaling pathway (**Figure 6A–6B**). However, the leading-edge subsets of genes were quite different for these 2 factors (**Figure 6E**). For example, Ascl1 activated expressions of the receptor encoding genes, *NOTCH1* and *NOTCH3*, while Neurog2 enhanced the expression of a different branch of the Notch signaling pathway, such as the *JAG1* ligand encoding gene. Moreover, Neurog2 and Ascl1 showed opposite regulations of the Hedgehog signaling pathway; Neurog2 activated, while Ascl1 inhibited the Hedgehog pathway (**Figure 6C–6D**). The heat map of the leading-edge subsets of genes confirmed this divergence (**Figure 6F**). It is worth noting that due to the bulk effect of RNA-seq from a mixture of both converted neurons and non-converted glioma cells, the gene set related to cell cycle did not significantly change (**Supplementary Figure S12**). Together, the transcriptome analyses confirmed the neuronal fate commitment during early expression of Neurog2, and suggested divergent molecular mechanisms between Neurog2 and Ascl1 in converting GBM cells into neurons.

### *In vivo* neuronal conversion of human GBM cells in a xenograft mouse model

**Figure 7:**
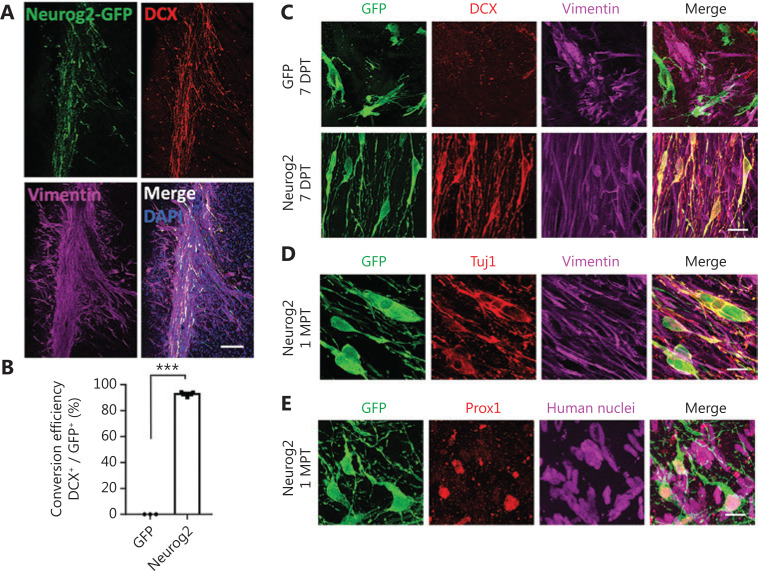
*In vivo* neuronal conversion of human glioblastoma (GBM) cells in a xenograft mouse model. (A) Representative images illustrating the transplanted human U251 GBM cells (mixed with CAG: Neurog2-IRES-eGFP retroviruses) in the striatum of Rag1^-/-^ immunodeficient mice. Note that most of the transplanted U251 cells (vimentin, magenta) transduced by Neurog2-GFP (green) retroviruses were immunopositive for neuronal marker DCX (red). Samples were taken at 3 weeks after transplantation. (B) Quantitative analyses of the conversion efficiency at 3 weeks after transplantation. Data are represented as the mean ± SEM and analyzed by Student’s *t*-tests. ^***^P < 0.001; *N* = 3 animals. Note that the conversion efficiency *in vivo* was also very high (92.8% ± 1.2%, DCX^+^/GFP^+^). (C) High magnification images showing that most of the transplanted U251 cells (vimentin, magenta) infected by Neurog2-GFP (green, bottom row) retroviruses were converted into neurons (DCX, red) as early as 1 week after transplantation. (D, E) Representative images showing the Neurog2-converted neurons (green) *in vivo*, expressing neuronal markers Tuj1 (red in D) and Prox1 (red in E) at 1 month after transplantation. Transplanted U251 human GBM cells were labeled by vimentin (magenta, D) and human nuclei (magenta, E). Scale bars, 200 μm in (A), 20 μm in (C)–(E).

**Figure 8:**
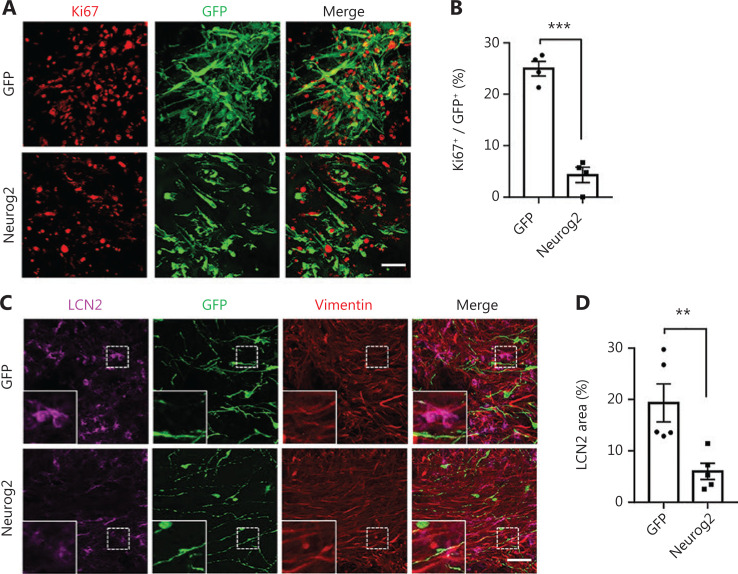
Proliferation arrest and amelioration of reactive astrocytes during *in vivo* neuronal conversion of glioblastoma (GBM) cells. (A, B) Representative images (A) and quantitative analyses (B) of proliferating U251 GBM cells (Ki67^+^, red) at 7 days after transplantation. Note a significant reduction of proliferation in Neurog2-converted cells. Scale bars, 50 μm. *N* = 4 animals. (C, D) Representative images (C) and quantitative analyses (D) of reactive astrocytes (labeled by LCN2, magenta) in the transplantation site with Neurog2-GFP or control GFP retroviral transduction (green fluorescent protein, green). Samples were taken at 3 weeks after transplantation. Scale bars, 50 μm. *N* = 5 animals. Data are represented as the mean ± SEM and analyzed using Student’s *t*-test. ^**^P < 0.01; ^***^P < 0.001.

Because the *in vitro* cell culture is very different from the *in vivo* environment inside the brain, we next determined the *in vivo* conversion efficiency of human GBM cells in a mouse brain model. To reduce complications from immune rejection, we performed intracranial transplantation of human GBM cells (5 × 10^5^ U251 cells) into the striatum bilaterally in Rag1^-/-^ immunodeficient mice (**Figure 7A**). Neurog2-GFP or control GFP retroviruses with the same volume (2 µL) and titer (2 × 10^5^ pfu/mL) were injected in each side of the striatum together with the transplanted GBM cells. Transplanted GBM cells were identified by vimentin (**Figure 7A**) or human nuclear staining (**Figure 7D**). Neurog2 overexpression (**Figure 7A**, green cells showing the Neurog2-GFP infected GBM cells) led to an efficient neuronal conversion, indicated by immature neuronal marker DCX staining (**Figure 7A–7C**, quantified in **7B**: Neurog2, 92.8% ± 1.2%, DCX^+^/GFP^+^, 3 weeks post transplantation, *N* = 3 mice). Other neuronal makers such as Tuj1 and Prox1 were also detected in the Neurog2-converted neurons at 1 month after transplantation (**Figure 7D–7E**). Importantly, consistent with our *in vitro* study, the cell proliferation among Neurog2-infected GBM cells significantly decreased when compared to the GFP control (**Figure 8A–8B**). Unexpectedly, we observed many LCN2-positive reactive astrocytes in brain areas transplanted with GBM cells, indicating neuroinflammation after cell transplantation. However, compared to the control group, Neurog2 overexpression in the transplanted GBM cells significantly reduced the number of reactive astrocytes (**Figure 8C–8D**), suggesting that neuronal conversion of GBM cells might ameliorate neuroinflammation in local transplantation areas.

In summary, human glioblastoma cells were efficiently reprogrammed into neuron-like cells through *in vivo* ectopic expression of the Neurog2 neural transcription factor in a xenograft mouse model. Moreover, this reprogramming approach significantly inhibited the proliferation of glioma cells and reduced reactive astrogliosis.

## Discussion

In this study, we showed that human GBM cells can be converted into terminally differentiated neurons by ectopic expression of a single neural transcription factor such as Neurog2, NeuroD1, or Ascl1. Remarkably, the neuronal conversion efficiency was high for all 3 factors, with Neruog2 achieving more than 90% conversion efficiency, both *in vitro* and *in vivo*. More importantly, we found that during neuronal conversion of GBM cells, Neurog2 and NeuroD1 yielded more glutamatergic neurons, while Ascl1 favored GABAergic neuron generation. RNA-seq analyses confirmed the early neuronal fate commitment induced by Neurog2 and resulted in divergent signaling pathways regulated by Ascl1 and Neurog2. The different neuronal subtypes induced by different neural transcription factors suggested that this cell conversion approach may have the potential to treat gliomas in different brain regions enriched with different subtypes of neurons.

### Transcriptome changes in response to neural transcription factor overexpression

We conducted RNA-seq to elucidate the transcriptome changes induced by neural transcription factors in human glioma cells. Several major findings emerged from these transcriptome analyses. First, we discovered significant activation of neuronal genes induced by Neurog2 in human glioblastoma cells. Most of the top upregulated DEGs encoded neuronal transcription factors or well-known regulatory factors involved in neurogenesis, confirming a critical role of Neurog2 as a pioneer factor in neurogenesis. Second, Ascl1 and Neurog2 triggered distinct transcriptional changes in human glioma cells, which may have explained different neuronal fates after conversion. During embryonic development, Neurog2 and Ascl1 are involved in the generation of different neuronal subtypes in distinct regions. Neurog2 regulates the generation of glutamatergic neurons in the dorsal telencephalon, while Ascl1 is more closely related to interneuron generation in the ventral region^36^. Consistent with their developmental functions, our transcriptome results showed that Ascl1 and Neurog2 elicited different neurogenic programs in human glioblastoma cells. Similar patterns were also found by other groups in neuronal conversion of human or mouse somatic cells^35,45^. For example, Masserdotti et al.^35^ reported that after ectopic expression in astrocytes, Neurog2 activated neuronal genes involved in glutamatergic neuron maturation such as INSM1 and NeuroD4, which were also found exclusively upregulated by Neurog2, but not Ascl1, during our human GBM cell conversion. In contrast, Ascl1 initially triggered a much broader developmental regulation program compared with the specific neuronal network activation by Neurog2. Therefore, there appeared to be a conservative mechanism involving different transcription factors, which had major roles in the determination of neuronal fate during neural differentiation or neural reprogramming.

### The advantages of cell conversion technology in treating GBM

GBM is an aggressive cancer with highly penetrative behavior and resistance to conventional therapeutic treatments, including CART therapy^5–7^. Traditional cancer treatment mainly aims to induce cell death, but such a “cancer-killing” strategy typically produces detrimental side effects on normal cells, including epithelial cells and immune cells. The severe side effects of current chemotherapy negatively impact the quality of life of cancer patients struggling to recover from the disease and arduous treatment regimens. Converting cancer cells into non-cancerous cells is a novel therapeutic strategy that may circumvent the severe side effects of current chemotherapy or radiation therapy on normal cells^13–18^. We showed that ectopic expression of several different neuronal transcription factors effectively converted GBM cells into non-proliferating neurons with functional properties. Moreover, tumor cell proliferation was significantly reduced after neuronal conversion, suggesting that cancer cell conversion may be a promising strategy to control glioma. The most significant advantage of our cancer cell conversion technology involves the minimal side effects on normal cells. One can envision that for small gliomas identified by magnetic resonance imaging or other brain imaging techniques, viruses expressing neuronal transcription factors such as Neurog2 or NeuroD1 could be injected directly into the tumor to induce neuronal conversion and inhibit glioma cell proliferation. For larger size gliomas, a surgical resection may be necessary, followed by injection of viruses to convert the remaining glioma cells into neurons. This approach would enable the neurosurgeons to preserve as much healthy brain tissue as possible to minimize collateral damage to the brain. More importantly, such injections into the glioma using viral particles would have minimal side effects on normal cells such as epithelial cells or immune cells, allowing recovering patients to live a relatively normal life.

### Challenges of cell conversion technology in cancer treatment

Like any new technology, there are many challenges facing the cancer cell conversion approach. While our data suggested that cancer cell conversion therapy has the potential to slow tumor growth, an obvious concern is that, although we achieved high conversion efficiency (over 90% in this study), the remaining cancer cells may still pose the threat of relapse. Nevertheless, we believe such cancer cell conversion technology can significantly delay the cancer development and extend the life span of cancer patients. Also of concern is that, while retroviruses may target rapidly proliferating cancer cells, the immunoreactivity of retroviruses is problematic when considering clinical trials^46–48^. The use of adeno-associated virus, which is a less immunogenic viral vector^49^, needs to be further investigated as an effective delivery system to introduce neuronal transcription factors into rapidly dividing GBM cells. A third challenge is to specifically targeting cancer cells, but not other types of cells in the body, for the expression of neuronal transcription factors necessary for cell conversion. Obviously, more work is needed to further address these challenges.

## Conclusions

In summary, our study showed an efficient neuronal conversion of U251 human GBM cells by forced overexpression of single neuronal transcription factor Neurog2, NeuroD1, or Ascl1. In addition, we found that this approach had significant potential in generating specific types of neurons using different factors. For example, Neurog2 and NeuroD1 yielded more glutamatergic neurons, while Ascl1 favored GABAergic neuron generation. More importantly, the neuronal conversion approach resulted in a significant proliferative arrest in both cultured glioblastoma cells and a glioma xenograft mouse model. These synergistic effects of neuronal conversion plus proliferation arrest suggested a potential new therapeutic strategy to treat brain cancer. While more studies are necessary to perfect this new technology, we envision that this unique approach of neuronal reprogramming may significantly benefit cancer patients in the future.
