# Supplementary material for: Transcription factor-based gene therapy to treat glioblastoma through direct neuronal conversion
Source: Cancer Biol Med. 2021 Aug 15;18(3):860–74. doi: 10.20892/j.issn.2095-3941.2020.0499 (PMC8330525; doi:10.20892/j.issn.2095-3941.2020.0499)
Supplement: Supplementary file 1 [file cbm-18-860-s001.pdf]

Supplementary materials

Plasmid construction and retrovirus production

The mouse NeuroD1 plasmid was constructed from our PCR product according to a template of the pAd NeuroD-I-nGFP[50] (Addgene, Watertown, MA, USA) and inserted into a pCAG-GFP-IRES-GFP retroviral vector[51] (a gift from Dr. Fred Gage) to generate pCAG-NeuroD1-IRES-GFP. Viral particles were packaged in gpg helper-free human embryonic kidney (HEK) cells to generate vesicular stomatitis virus glycoprotein (VSV-G)-pseudo typed retroviruses encoding neurogenic factors using a CellMax hollow fiber cell culture system (Spectrum Laboratories, Milpitas, CA, USA). The titer of GFP, Neurog2-GFP, NeuroD1-GFP, and Ascl1-GFP viral particles in the virus-containing medium was approximately 1×10^3 pfu/mL, 1×10^5 pfu/mL, 1×10^4 pfu/mL, and 9×10^4 pfu/mL respectively. The titer of GFP and Neurog2-GFP viral particles in the concentrated viruses was approximately 2×10^5 pfu/mL and 1×10^7 pfu/mL, respectively. The viral titer was determined using human kidney cell transduction. For *in vitro* infection, approximately 500 μL GFP, 5 μL Neurog2-GFP, 50 μL NeuroD1-GFP, and 5 μL Ascl1-GFP virus-containing medium was added to each well (24-well plates) to achieve comparable titers. For *in vivo* experiments, 2 μL GFP or 2 μL Neurog2-GFP (50× dilution) concentrated retroviruses were mixed with human GBM cells for transplantation, respectively.

Immunocytochemistry

Cultured cells were fixed in 4% paraformaldehyde (PFA) in phosphate-buffered saline (PBS) for 15 min at room temperature. The cells were washed 3 times using PBS and incubated in blocking buffer (5% normal donkey serum, 0.05% Triton X-100 in PBS) for 40 min. The cells were then incubated with primary antibodies in blocking buffer overnight at 4 °C. The next day, the cells were washed 3 times by 0.05% Triton X-100 in PBS and incubated with appropriate secondary antibodies conjugated to Alexa Fluor 488, Alexa Fluor 546, or Alexa Fluor 647 (1:1,000; Molecular Probes, Eugene, OR, USA) for 1 h at room temperature. After 3 washes with 0.05% Triton X-100 in PBS, the coverslips were mounted onto microscope slides (75 × 25 × 1 mm; VWR, Radnor, PA, USA) with a mounting solution containing 4′,6-diamidino-2-phenylindole (Invitrogen). The slides were first examined with a revolve microscope (Echo, Revolve R4; VWR) and further analyzed with a confocal microscope (LSM 800; Zeiss, Jena, Germany). The images were acquired and analyzed using Zeiss software (Zeiss). Antibody information was collected in **Supplementary Table S1**.

RNA isolation, reverse transcription, and RT-PCR

RNA isolations from cultured cells were performed at desired time points using a NucleoSpin® RNA kit (Macherey-Nagel) following the manufacturer's protocols. Reverse transcription was performed using 5× qScriptTM cDNA SuperMix (Quanta Biosciences, Beverly, MA, USA) from isolated RNA samples. PerfeCTaTM SYBR® Green SuperMix, ROXTM (Quanta Biosciences) was used for RT-PCR. Glyceraldehye 3-phosphate dehydrogenase was used as the internal control. Each sample had 3 replicates for each target. The sequences of all primers were listed in **Supplementary Table S2**.

Western blot

Cells were lysed, fractionated by 10% SDS-Tris glycine, and transferred to a 45 μm polyvinylidene difluoride membrane. The membranes were blocked and incubated with the primary antibodies against GSK3β (27C10) (rabbit, 1:1,000; 9315; Cell Signaling Technology, Danvers, MA, USA), and glyceraldehyde 3-phosphate dehydrogenase (rabbit; 1:5,000; G9545; Sigma-Aldrich) at 4 °C overnight. After washing, it was incubated with secondary antibodies (1:15,000; goat anti-Rb 800; 925-32210; P/N). Scanning was performed using an Odyssey Clx (LI-COR, Lincoln, NB, USA).

Patch clamp recordings of cultured cells

For the converted neurons, whole-cell recordings were performed using a Multiclamp 700A patch clamp amplifier (Molecular Devices, Palo Alto, CA, USA) as previously described,[52] and the chamber was constantly perfused with a bath solution consisting of 128 mM NaCl, 30 mM glucose, 25 mM HEPES, 5 mM KCl, 2 mM $CaCl_2$, and 1 mM $MgCl_2$. The pH of the bath solution was adjusted to 7.3 with NaOH, and the osmolarity was at 315–325 mOsm/L. Patch

Table S1 Antibodies used for immunostaining

Antibodies	Species	Dilution	Company	Catalog No.
Polyclonal anti-NEUN	Guinea pig	1:1,000	Millipore	ABN90P
Polyclonal anti-MAP2	Rabbit	1:2,000	Millipore	AB5622
Polyclonal anti-MAP2	Chicken	1:2,000	Abcam	AB5392
Monoclonal anti-Tuj1	Mouse	1:1,000	COVANCE	MMS-435P
Polyclonal anti-Tbr1	Rabbit	1:600	Abcam	AB31940
Polyclonal anti-Prox1	Rabbit	1:1,000	ReliaTech GmbH	102-PA32
Polyclonal anti-FoxG1	Goat	1:600	Abcam	AB3394
Monoclonal anti-Ctip2	Rat	1:600	Abcam	AB18465
Polyclonal anti-VGluT1	Rabbit	1:1,000	Synaptic Systems	135302
Polyclonal anti-SV2	Mouse	1:1,000	DSHB	SV2
Polyclonal anti-GFP	Chicken	1:1,000	Abcam	AB13970
Polyclonal anti-GFAP	Chicken	1:1,000	Millipore	AB5541
Polyclonal anti-GFAP	Rabbit	1:1,000	Millipore	AB5804
Monoclonal anti-Human Nuclei (HuNu)	Mouse	1:1,000	Millipore	MAB1281
Monoclonal anti-S100β	Mouse	1:1,000	Abcam	AB66028
Polyclonal anti-DCX	Goat	1:500	Santa Cruz	SC-8066
Polyclonal anti-SOX2	Rabbit	1:1,000	Millipore	AB5603
Polyclonal anti-Ki67	Rabbit	1:1,000	Abcam	AB15580
Monoclonal anti-BrdU	Rat	1:1,000	Accurate	OBT0030
Polyclonal anti-GABA	Rabbit	1:1,000	Sigma	A2052
Monoclonal anti-GM130	Mouse	1:800	BD	610822
Polyclonal anti-ATG5	Rabbit	1:600	Novus	NB110-53818
Polyclonal anti-EGFR	Rabbit	1:600	Santa Cruz	SC-1005
Monoclonal anti-Nestin	Mouse	1:800	Neuromics	MO15012
Polyclonal anti-IL13Ra2	Goat	1:600	R&D	AF146
Polyclonal anti-Olig2	Rabbit	1:600	Millipore	AB9610
Polyclonal anti-GAP43	Rabbit	1:800	Abcam	AB16053
Polyclonal anti-Connexin 43	Rabbit	1:800	Abcam	AB11370
Monoclonal anti-GSK3β	Rabbit	1:800	Cell Signaling	9315
Polyclonal anti-Vimentin	Rat	1:1,000	R&D	MAB2105
Monoclonal anti-NeuroD1	Mouse	1:1,000	Abcam	AB60704
Polyclonal anti-Neurog2	Rabbit	1:600	Abcam	AB154293
Polyclonal anti-Ascl1	Rabbit	1:800	Abcam	AB74065

Table S2 Sequences of primers used in RT-PCR.

Primers	Sequence
DCX-F	TGCTTGGGCCTCACACTAGC
DCX-R	CATATACCGCAATCAAGGAAATACTC
Ascl1-F	GTCAAGTTGGTCAACCTGGG
Ascl1-R	CTCATCTTCTTGTTGGCCGC
NEUROD2-F	TCAGACATGGACTATTGGCAG
NEUROD2-R	GGGACAGGAAAGGGAACC
NEUROG2-F	ATTTGCAATGGCTGGCATCT
NEUROG2-R	CACAGCCTGCAGACAGCAAT
NEUROD1-F	CCTGCAACTCAATCCTCGGA
NEUROD1-R	GGCATGTCCTGGTTCTGCTC
GAPDH-F	TGGGCTACACTGAGCACCAG
GAPDH-R	GGGTGTCGCTGTTGAAGTCA
ASCL1-F	CAACGACTTGAACTCCATGGC
ASCL1-R	TTGGTGAAGTCGAGAAGCTCC
DLX2-F	CAACAACGAGCCTGAGAAGGAG
DLX2-R	GGAAACTGGAGTAGATGGTGCG

pipettes were pulled from borosilicate glass (3–5 MΩ) and filled with a pipette solution consisting of 135 mM KCl, 5 mM Na-phosphocreatine, 10 mM HEPES, 2 mM EGTA, 4 mM MgATP, and 0.5 mM Na_2GTP (pH 7.3, adjusted with KOH). The series resistance was typically 10–30 MΩ. For voltage-clamp experiments, the membrane potential was typically held at −70 or −80 mV. Data were acquired using pClamp 9 software (Molecular Devices), sampled at 10 kHz, and filtered at 1 kHz. The Na+ and K+ currents and action potentials were analyzed using pClamp 9 Clampfit software. Spontaneous synaptic events were analyzed using MiniAnalysis software (Synaptosoft, Decator, GA, USA). All experiments were conducted at room temperature.

Mitochondrial tracker incubation

MitoTracker™ Red CMXRos (Invitrogen) was used to show mitochondrial morphology and distribution. MitoTracker was diluted with culture medium to a final concentration of 500 nM. The cells were incubated with MitoTracker for 1 h and then fixed with 4% PFA. This was followed by the regular immunohistochemistry protocol.

BrdU labeling and cell proliferation assays

Cell proliferation was examined by BrdU incorporation. BrdU was added in cell culture medium (10 mM) at indicated durations. At the desired time points, the cells were fixed in 4% PFA for 15 min, and then treated with 2 M HCl for 1 h at room temperature, and washed by PBS, 3 times, with 5 min each time. This was followed by blocking and sequential incubations with anti-BrdU antibody (1:1,000; Accurate Chemical, Westbury, NY, USA) and corresponding secondary antibody.

Data and statistical analysis

Cell counting and the fluorescence intensity were performed in a single blind manner with randomly chosen fields of randomly chosen pictures and analyzed by ImageJ software (National Institutes of Health, Bethesda, MD, USA). Data are represented as the mean ± SEM. Multiple group comparisons were performed using two-way analysis of variance followed by Dunnett's tests. Two group comparisons were performed using Student's *t*-test.

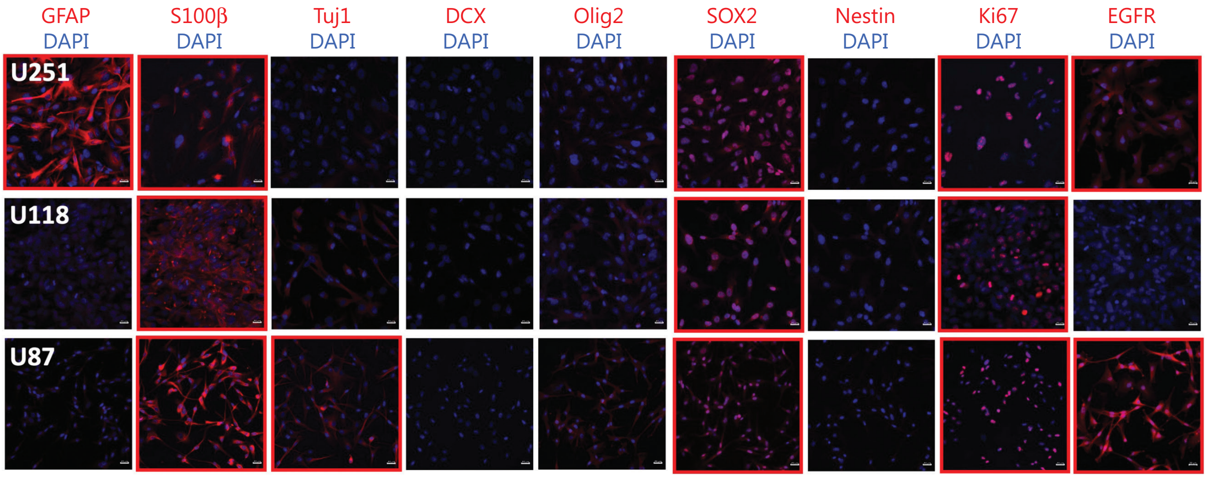

Figure S1 Characterization of glioblastoma (GBM) cell lines. Representative images showing the immunohistochemistry screening results (red) in U251, U118, and U87 human GBM cells (4′,6-diamidino-2-phenylindole, blue). Red boxes indicate positive signals. Glial fibrillary acidic protein (GFAP), a reactive astroglia marker; S100β, astroglial marker; Tuj1, DCX, immature neuronal markers; Sox2, stem cell marker; Nestin, stem/progenitor cell marker; Olig2, oligodendrocyte marker; Ki67, proliferation marker; EGFR, cancer marker. Scale bars represent 50 μm.

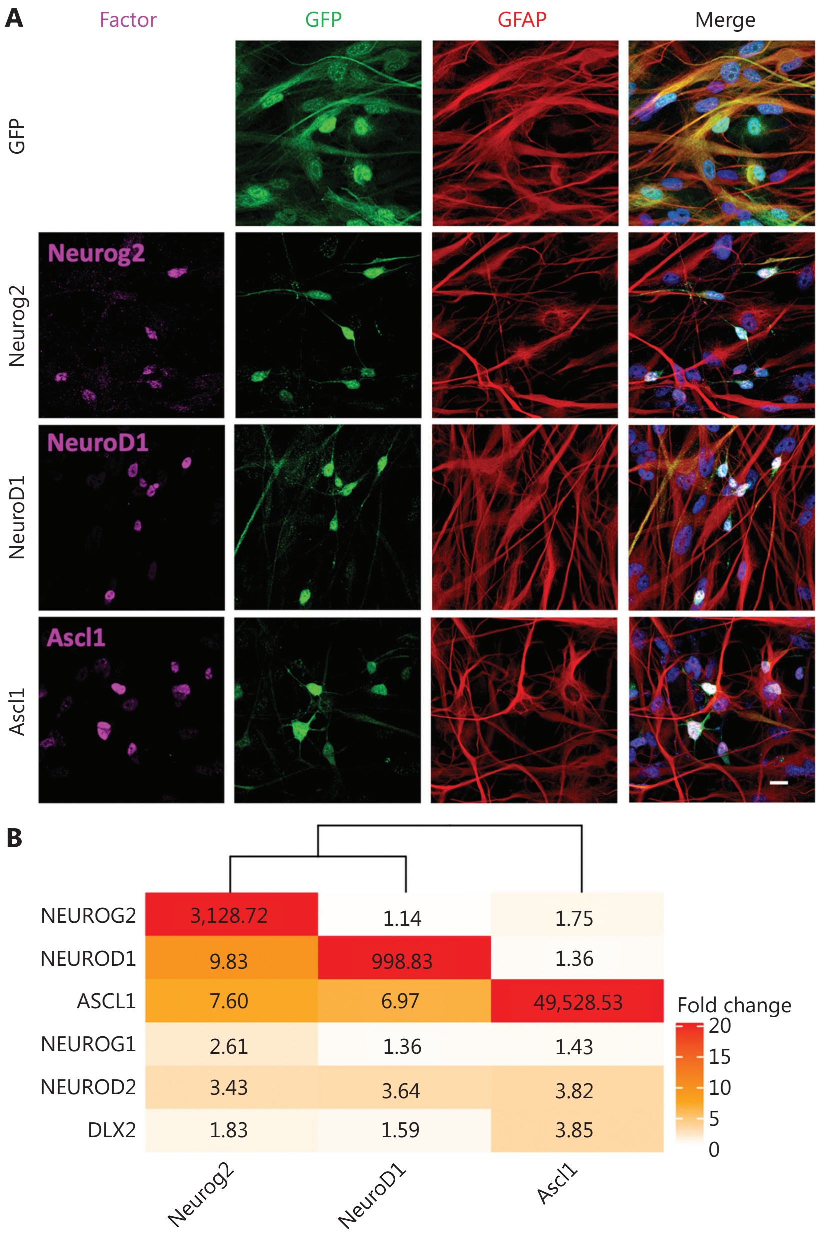

Figure S2 Overexpression of neural transcription factors, Neurog2, NeuroD1, or Ascl1, in human glioblastoma (GBM) cells. (A) Representative images showing the immunostaining of Neurog2, NeuroD1, or Ascl1 in U251 human GBM cells after retroviral transduction at 20 dpi. Scale bars, 20 µm. (B) A heat map summarizing the expression of different neural transcription factors revealed by real-time qPCR. Note a huge transcriptional increase of NEUROG2, NEUROD1 or ASCL1 in the corresponding group. Data were normalized to control green fluorescent protein-expressing U251 cells. Data are represented as the mean from N = 3 batches of cultures. Samples were collected at 20 dpi.

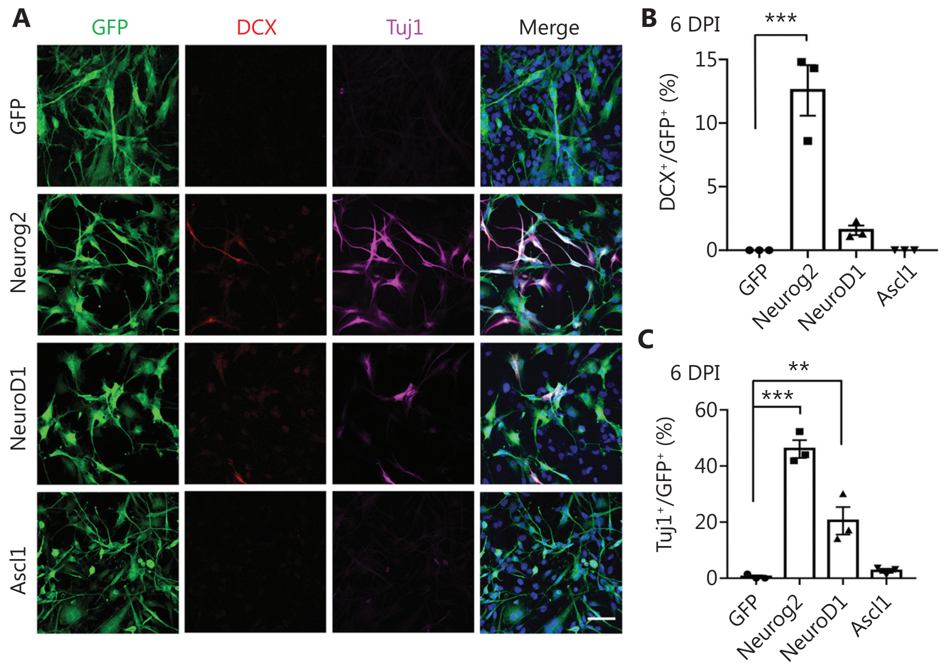

Figure S3 Rapid neuronal conversion of human glioblastoma (GBM) cells by Neurog2 and NeuroD1. (A) Representative images showing the immunostaining of immature neuronal markers Doublecortin (DCX, red) and β3-tubulin (Tuj1, magenta) in U251 human GBM cells with retroviral expression of Neurog2, NeuroD1, Ascl1, or GFP alone at 6 dpi. Scale bars, 50 μm. (B, C) Quantitative analyses of the conversion efficiency at 6 dpi. Note that Neurog2 and NeuroD1 overexpressions induced a fast production of DCX^+ cells (B) and $Tuj1^+$ cells (C). Data are represented as the mean ± SEM and analyzed by one-way analysis of variance followed by Dunnett's test. $^{**}P < 0.01$; $^{***}P < 0.001$; $N > 200$ cells from triplicate cultures.

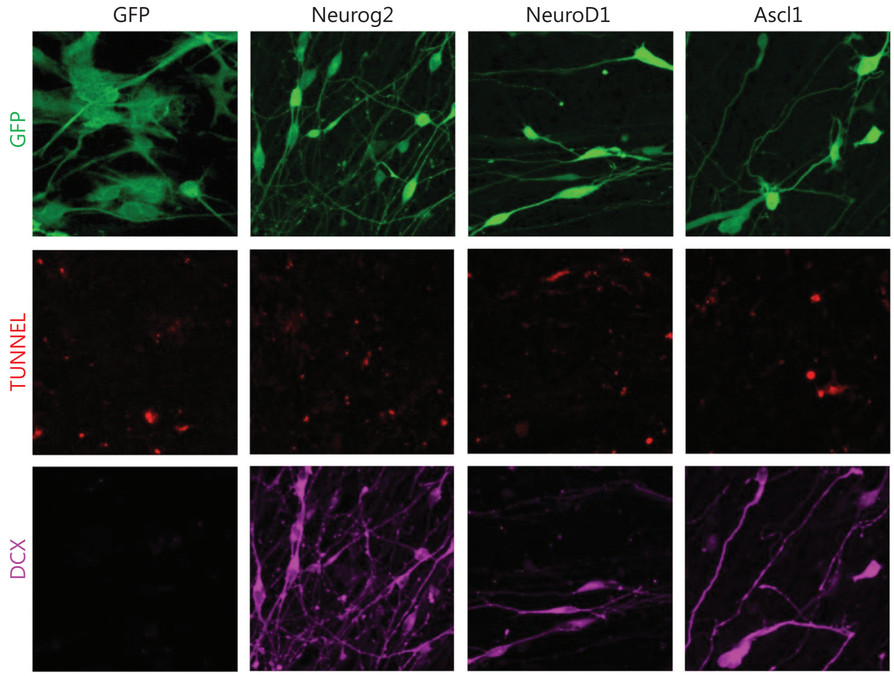

Figure S4 Investigation of cell apoptosis during conversion. Representative images showing comparable low apoptosis among control and experimental groups (TUNEL staining, red). Samples were collected at 25 days after infection in U251 human glioblastoma cells.

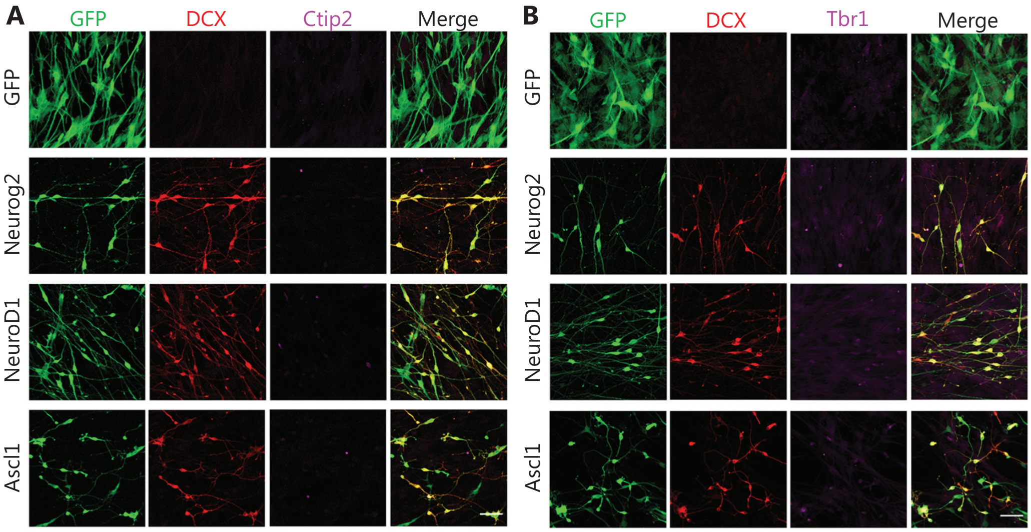

Figure S5 Characterization of the converted neurons from human glioblastoma (GBM) cells. (A, B) Representative images showing the immunostaining of cortical neuron marker, Ctip2 (magenta, A), or Tbr1 (magenta, B) in U251 human GBM cells overexpressing Neurog2, NeuroD1, Ascl1, or GFP alone. Samples were collected at 20 dpi. Scale bars, 50 µm.

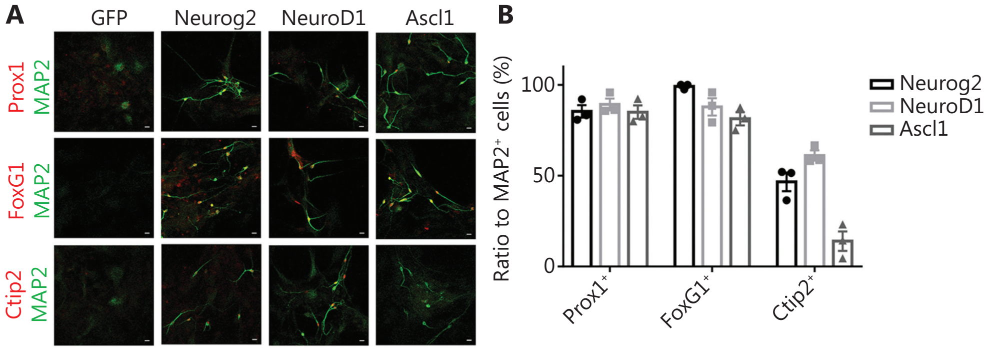

Figure S6 Characterization of the converted neurons from human astrocytes. (A) Representative images showing the expression of neuronal subtype markers in converted neurons from human astrocytes (HA1800 cells; ScienCell). Most of the Neurog2-, NeuroD1-, and Ascl1-converted neurons (MAP2, green) were immunopositive for hippocampal neuronal marker Prox1 (red, top row) and forebrain marker FoxG1 (red, second row). Note that there were a reasonable number of Ctip2$^+$ neurons (red, third row) converted by Neurog2 or NeuroD1. Scale bars, 20 µm. (B) Quantitative analyses of Neurog2-, NeuroD1-, and Ascl1-converted neurons from human cortical astrocytes (HA1800 cells; ScienCell). Samples were at 30 dpi. Data are represented as the mean ± SEM. *N* > 50 cells from triplicate cultures.

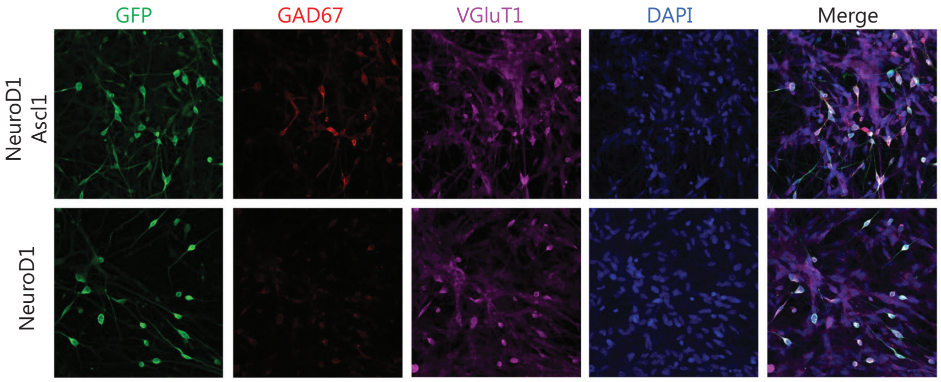

Figure S7 Co-expression of Ascl1 and NeuroD1 in human glioblastoma cells. Sample images showing the neuronal subtypes (GAD67 for GABAergic neurons, red; VGluT1 for glutamatergic neurons, magenta) converted by co-expression of Ascl1 and NeuroD1 (upper panel) and compared with the results from NeuroD1 alone (lower panel). Samples were at 30 dpi.

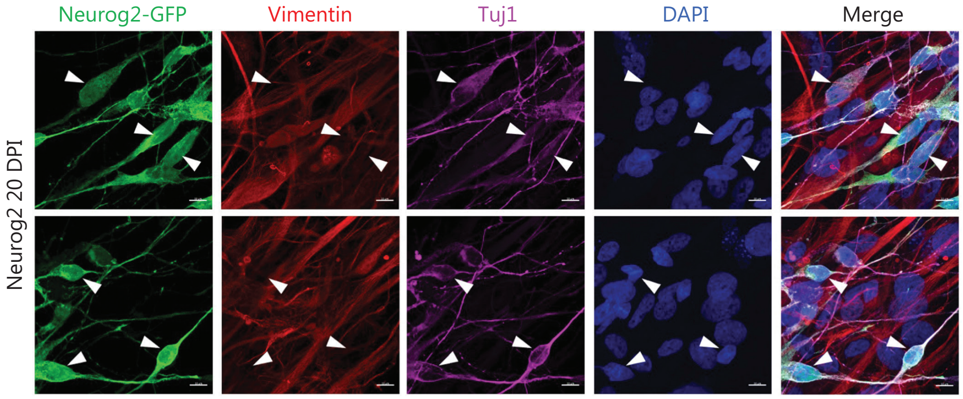

Figure S8 Downregulation of glial markers during neuronal conversion of human GBM cells. Sample images from 2 different image fields showing that Neurog2 overexpression (green fluorescent protein, green) led to neuronal conversion (Tuj1, magenta) and downregulation of astrocyte marker vimentin (red). Arrowheads indicate the infected cells exhibiting neuronal marker, Tuj1, and downregulated vimentin. Samples were U251 human glioblastoma cells at 20 days after infection.

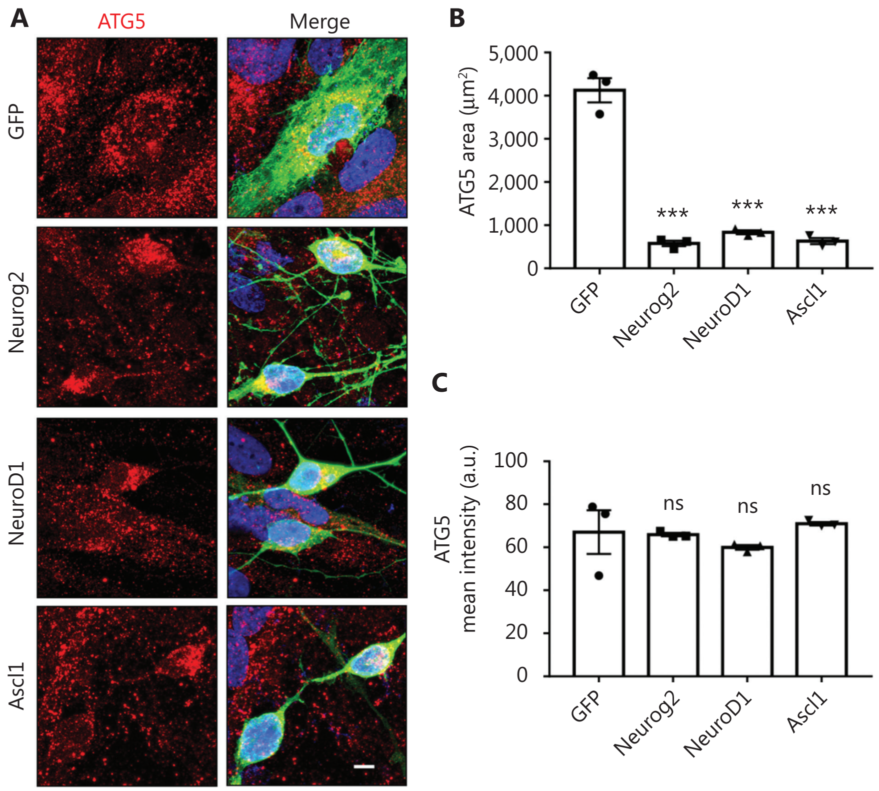

Figure S9 Examination of autophagy/lysosomes during neuronal conversion of human glioblastoma (GBM) cells. (A) Representative images illustrating the distribution and morphological changes of autophagy/lysosomes (ATG5, red) during neuronal conversion of U251 human GBM cells. Scale bars, 10 μm. (B, C) Quantification analyses of ATG5 covered area (B) and mean intensity (C) in transduced U251 cells at 30 dpi. $N \geq 150$ cells from triplicate cultures. Data are represented as the mean ± SEM and analyzed by one-way analysis of variance followed by Dunnett's test. $^{***}P < 0.001$.

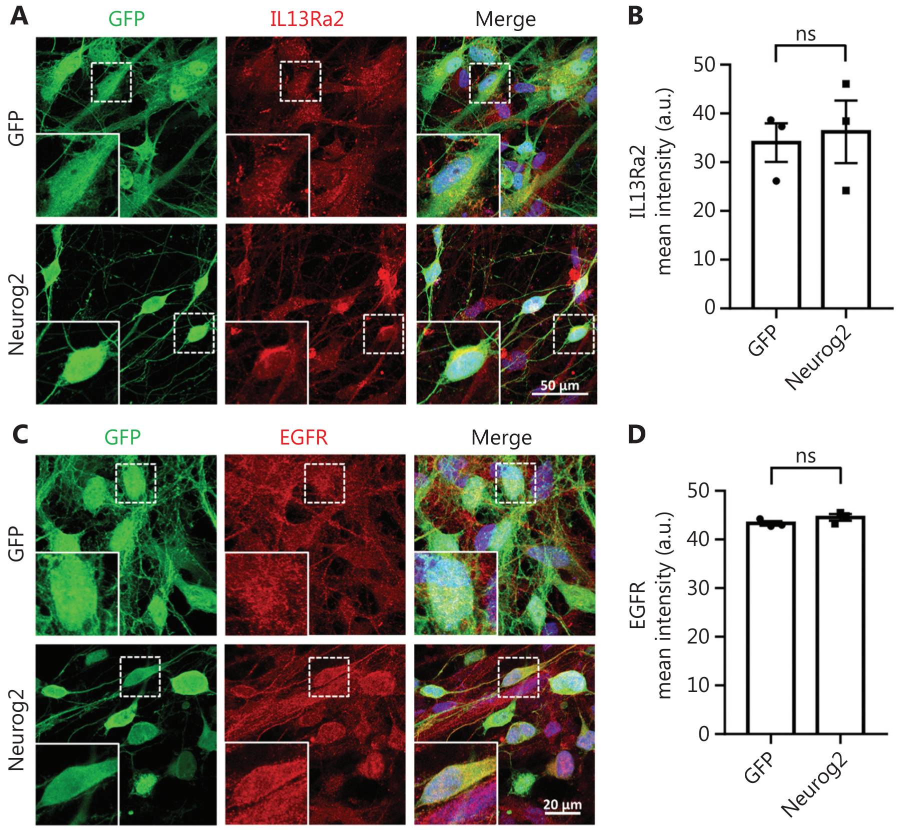

Figure S10 Investigation of cancer markers in Neurog2-converted neurons from human glioblastoma (GBM) cells. (A) Representative images showing the immunostaining of glioma restricted receptor, IL13Ra2 (red), in U251 human GBM cells expressing Neurog2 or GFP (green) at 20 dpi. Scale bars, 50 µm. (B) Quantitative analyses of IL13Ra2 mean intensity in transduced U251 cells at 20 dpi. (C) Immunostaining of general cancer marker, EGFR (red), in U251 human glioblastoma cells expressing Neurog2 or GFP (green) at 20 dpi. Scale bars, 50 µm. (D) Quantification analyses of EGFR mean intensity in transduced U251 cells at 20 dpi. Data are represented as the mean ± SEM and were analyzed by Student's *t*-test. $N \geq 40$ cells from triplicate cultures.

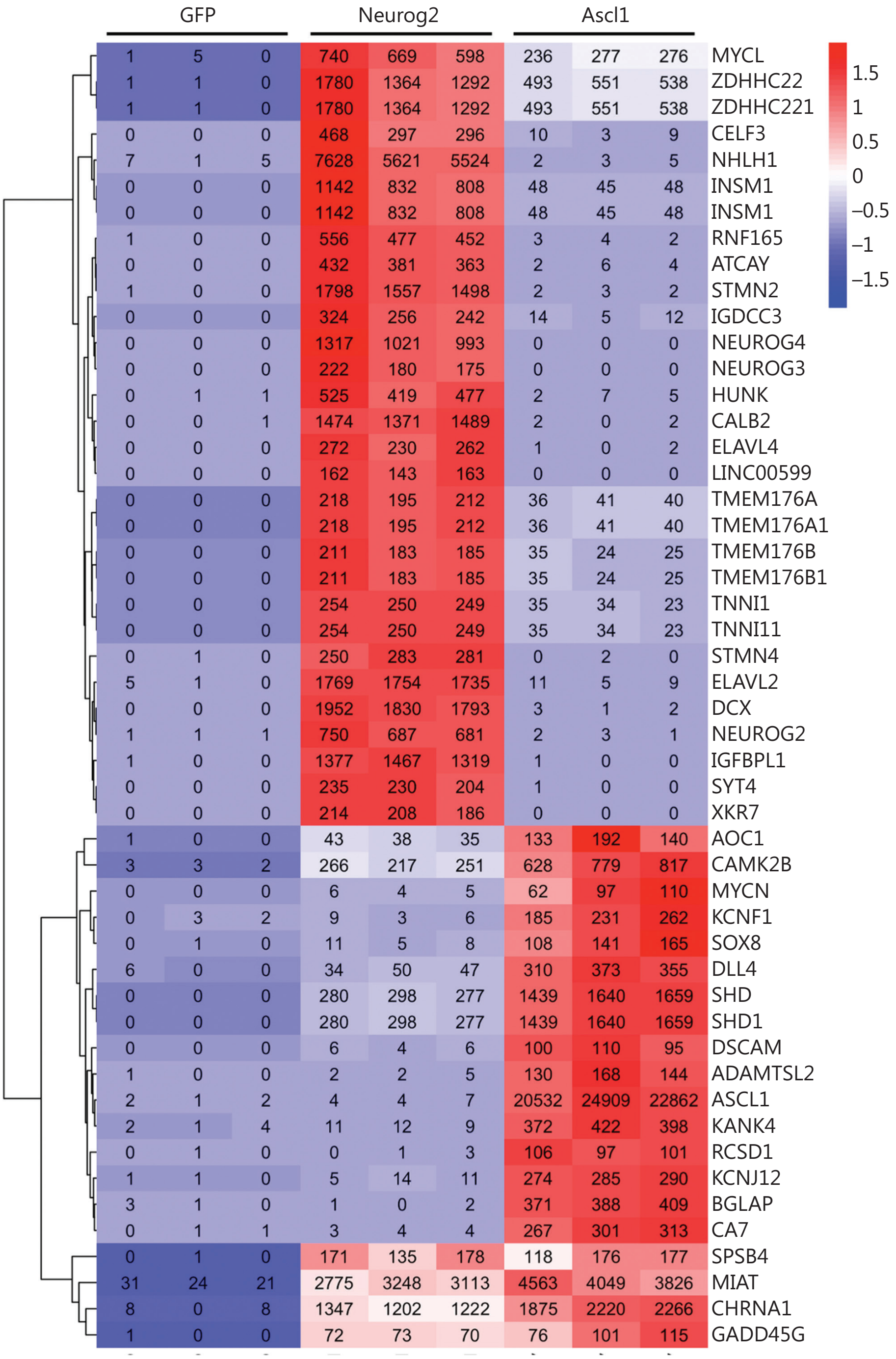

Figure S11 Top upregulated differentially-expressed genes (DEGs) in response to Neurog2 or Ascl1 overexpression in human glioblastoma cells. A heat map with hierarchical clustering showing the top 25 upregulated DEGs (sorted by fold change, > 100 normalized read counts in at least one sample) in response to Ascl1 or Neurog2 overexpression in U251 human glioblastoma cells. The color was scaled within each row. Normalized read count values are presented.

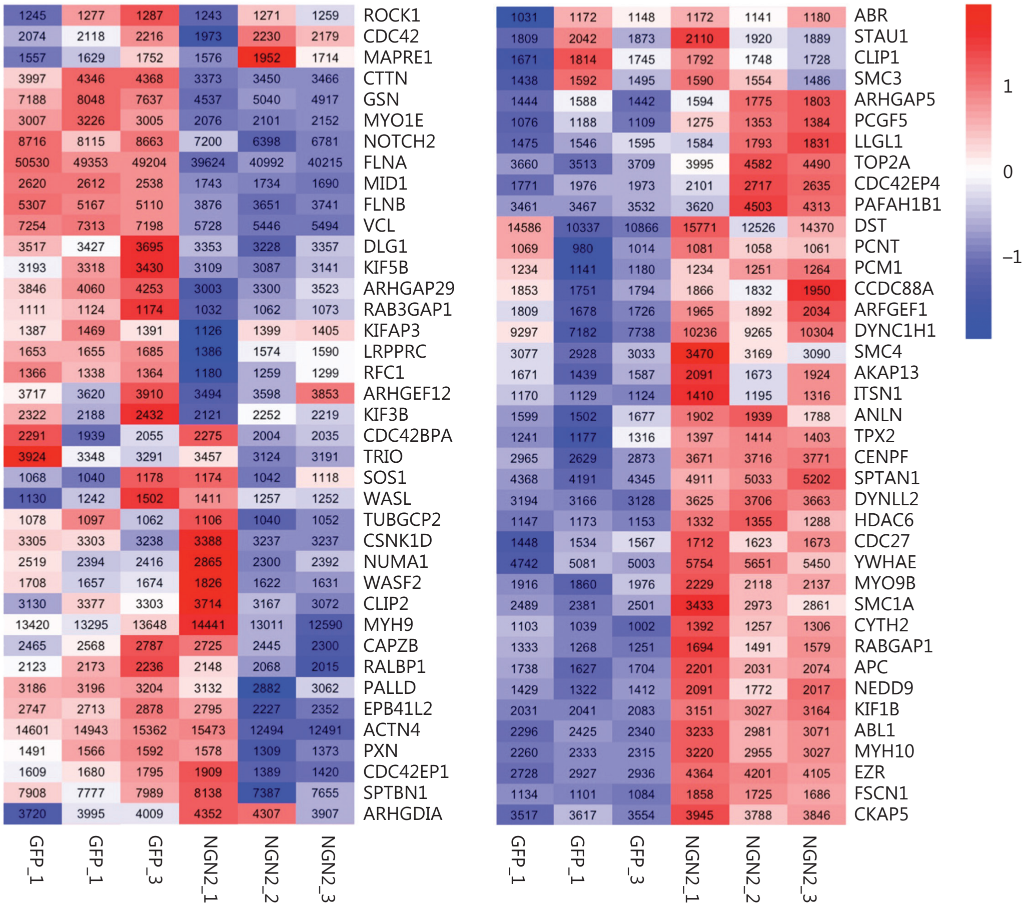

Figure S12 Investigation of the cell cycle gene set in response to Neurog2-induced neuronal conversion of human glioblastoma cells. Heat map illustrating the subsets of genes (read count > 1,000) during conversion corresponding to mitotic spindle hallmark in gene set enrichment analysis. Color scaled within each row. Red color indicates high expression level, while blue color indicates low expression level.
